# Crystal Lattice Recovery and Optical Activation of Yb Implanted into β-Ga_2_O_3_

**DOI:** 10.3390/ma17163979

**Published:** 2024-08-10

**Authors:** Mahwish Sarwar, Renata Ratajczak, Vitalii Yu. Ivanov, Sylwia Gieraltowska, Aleksandra Wierzbicka, Wojciech Wozniak, René Heller, Stefan Eisenwinder, Elżbieta Guziewicz

**Affiliations:** 1 Institute of Physics, Polish Academy of Sciences, Al. Lotnikow 32/46, 02-668 Warsaw, Poland; 2 National Centre for Nuclear Research, Soltana 7, 05-400 Otwock, Poland; 3 Helmholtz-Zentrum Dresden-Rossendorf, Institute of Ion Beam Physics and Materials Research, Bautzner Landstrasse 400, D-01328 Dresden, Germany

**Keywords:** Ga_2_O_3_, Yb, Rutherford backscattering, photoluminescence

## Abstract

β-Ga_2_O_3_ is an ultra-wide bandgap semiconductor (E_g_~4.8 eV) of interest for many applications, including optoelectronics. Undoped Ga_2_O_3_ emits light in the UV range that can be tuned to the visible region of the spectrum by rare earth dopants. In this work, we investigate the crystal lattice recovery of $\left(\overset{¯}{2}01\right)$-oriented β-Ga_2_O_3_ crystals implanted with Yb ions to the fluence of 1 ×10^14^ at/cm^2^. Post-implantation annealing at a range of temperature and different atmospheres was used to investigate the β-Ga_2_O_3_ crystal structure recovery and optical activation of Yb ions. Ion implantation is a renowned technique used for material doping, but in spite of its many advantages such as the controlled introduction of dopants in concentrations exceeding the solubility limits, it also causes damage to the crystal lattice, which strongly influences the optical response from the material. In this work, post-implantation defects in β-Ga_2_O_3_:Yb crystals, their transformation, and the recovery of the crystal lattice after thermal treatment have been investigated by channeling Rutherford backscattering spectrometry (RBS/c) supported by McChasy simulations, and the optical response was tested. It has been shown that post-implantation annealing at temperatures of 700–900 °C results in partial crystal lattice recovery, but it is accompanied by the out-diffusion of Yb ions toward the surface if the annealing temperature and time exceed 800 °C and 10 min, respectively. High-temperature implantation at 500–900 °C strongly limits post-implantation damage to the crystal lattice, but it does not cause the intense luminescence of Yb ions. This suggests that the recovery of the crystal lattice is not a sufficient condition for strong rare-earth photoluminescence at room temperature and that oxygen annealing is beneficial for intense infrared luminescence compared to other tested environments.

## 1. Introduction

β-gallium oxide (β-Ga_2_O_3_), an ultra-wide bandgap material, has attracted keen attention in the field of electronic and photonic research due to its wide bandgap of ~4.8 eV, which is higher than the most rigorously investigated semiconductors like GaN (3.4 eV) and ZnO (3.37 eV) [1,2,3]. The electric breakdown voltage of 8 MV/cm [4], higher than GaN (3.3 MV/cm) [5], is another interesting characteristic of this semiconductor and one that helps it to stand out amongst other semiconductors. Apart from that, thermal and chemical stabilities offer a variety of prospective applications in commercialized devices [6]. The main reported applications include solar-blind photodetectors [7,8], field effect transistors, air-purifiers, sensors, space communication [6,9], and UV transparent electrodes [10]. The mentioned unique properties of β-Ga_2_O_3_ allow all of these devices to operate at high temperatures, high voltages, and in highly radiative environments [6]. 

Additionally, β-Ga_2_O_3_ has also the predisposition to be a good host material for the multicolor optically active centers from the UV to IR region of the spectrum, including a number of rare-earth elements [11]. Although undoped β-Ga_2_O_3_ emits light in the UV and blue region [12], the optical tuning to obtain luminescence in other regions is vital for its optoelectronic applications. Ytterbium, being one of the rare-earth (RE) elements, exhibits narrow emission lines in IR region, and this luminescence is undisturbed by emissions from defects in the host material [13]. 

**Figure 1:**
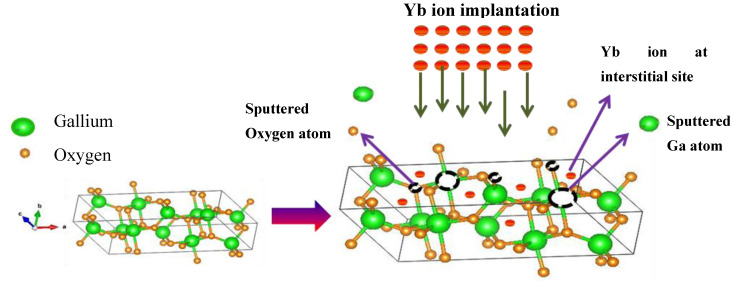
Schematic of β-Ga_2_O_3_ crystal structure and damaged β-Ga_2_O_3_ after Yb ion implantation with Yb on interstitial sites.

Ion implantation is one of the routine doping techniques used for the alteration of optical, electrical, and magnetic properties of semiconductors [14,15]. The benefits of this technique include the relatively low temperature for the doping, and the ability to control the distribution and precise dosage of dopants. However, the ballistic nature of this doping method indulges the formation of undesired defects (Figure 1), causing a change in the optical and electronic properties of β-Ga_2_O_3_ and decreasing its efficiency. Therefore, annealing aimed at the recovery of the crystal lattice damaged by implantation and the optical activation of the RE ion is required.

Different kinds of annealing have been reported to show the recovery of the crystal structure after irradiation, of which rapid thermal annealing (RTA) is evidently beneficial [13]. Some reports can be found in the literature about the defects created by ion implantation and luminescence of β-Ga_2_O_3_ implanted with different ions, but detailed studies of radiation-induced structural defects and particularly the optimization of annealing conditions for the considerable recovery of the crystal structure have not been found, despite the fact that it is crucial to obtain the optimum annealing conditions for prospective optoelectronic applications. A. Azarov et al. [16] reported on the room temperature implantation of β-Ga_2_O_3_ single crystals with a Ni ion fluence of 2 × 10^14^ at/cm^2^ followed by annealing in air for 30 min at 200–900 °C. Similar studies were performed by S. B. Kjeldby et al. [17]. Both reports showed that the highest temperature used led to partial crystal lattice recovery, but the out-diffusion of the dopant, whose prevention is crucial for an intensive optical response, was not studied. K. Lorenz et al. [18] investigated single Ga_2_O_3_ crystals implanted at 300 keV energy with europium (Eu) ion fluences ranging from 1 × 10^13^ to 4 × 10^15^ at/cm^2^. The RBS/c study revealed a complex mixture of structural defects caused by implantation. The crystal implanted with 4 × 10^15^ at/cm^2^ was treated with isochronous RTA in the Ar atmosphere for 30 s at temperatures ranging from 500 to 1200 °C. High-temperature annealing at 1100 °C was found to be efficient for the crystal structure recovery, but it had a side effect of Eu out-diffusion. This is not favorable from an application perspective, because the Eu^3+^ emission intensity then decreases. E. Wendler et al. [19] studied the ion implantation-induced effects in (010)-oriented bulk β-Ga_2_O_3_ crystals with P, Ar, and Sn ions for fluences ranging from 1 × 10^11^ to 2 × 10^15^ at/cm^2^. Damage peaks were evidently present in the RBS/c spectra, wherein the evolution of the damage peaks was observed with the increase in ion fluence. Point defects, their complexes, and damage clusters were associated with implantation. However, no method has been proposed to get rid of these defects or for effective crystal recovery accompanied by optical emission. 

This brief review of the progress in implanted β-Ga_2_O_3_ shows that intensive studies about the radiation-induced defects have been performed, but the understanding of the created defects and their evolution with thermal treatment to obtain luminescence is missing. The annealing conditions were not widely investigated, and the annealing time, the temperature, and the ambiance for the significant crystal recovery without the out-diffusion of the dopant have not yet been established. The main reason for this study is to fill this gap.

Our previous research has been primarily focused on the determination of defect concentrations as a function of ion fluence for Yb-implanted β-Ga_2_O_3_. It has been shown that for some fluences, important defect transformations occur in the implanted zone, and only defects created by low fluence can be removed via annealing [20]. In addition, it was also found that at the higher ion fluences used (above 1 × 10^14^ at/cm^2^ = 0.5 dpa), the crystal phase transition takes place. In contrast to other defects created at this stage of fluences, the phase transition can be reversed by using appropriate annealing conditions [16,20,21]. However, no optical investigations were reported in the previous studies. 

Therefore, to fill the aforementioned knowledge gap on the optimal annealing conditions for RE optical activation, we aim to study the evolution of defects and eventually the optical properties by using different temperatures and times, and by varying the ambiance of annealing. To achieve this goal, β-Ga_2_O_3_ crystals were implanted with Yb ions to the fluence 1 × 10^14^ at/cm^2^, which was previously established to be the limit after which the lattice recovery becomes annealing resistant [20]. Additionally, we have investigated the effect of high-temperature (HT) implantation. It has been reported that HT implantation can reduce the level of implantation-related damage due to efficient dynamic annealing [14,22,23]. M. Peres et al. [24] showed that the damage level of as-implanted β-Ga_2_O_3_:Eu samples is significantly lower when the implantation temperature is increased to 400–600 °C. Raising the implantation temperature above room temperature also resulted in increased optical activity of Eu^3+^ ions.

In the present study, the recovery of the crystal lattice and Yb sites positions was evaluated via RBS/c [25] and supported by McChasy, which is the Monte Carlo simulation code [26,27]. Consequently, the RBS/c results presented here may be considered as qualitative as well as quantitative. The high-resolution X-ray diffraction (HR XRD) results of the as-implanted and annealed samples are also shown. Furthermore, we present here the optical Yb^3+^ response to structural changes, with the aim of establishing the optimal annealing conditions for optoelectronic applications of β-Ga_2_O_3_:RE systems.

## 2. Materials and Methods

$\left(\overset{¯}{2}01\right)$-oriented β-Ga_2_O_3_ bulk single crystal wafers purchased from Novel Crystal Co. Technology (Tokyo, Japan) were used for the analysis. The wafers were diced into 1 × 1 cm pieces at the Institute of Physics, Polish Academy of Sciences (IP PAS). The diced crystals were implanted with Yb ions of 150 keV energy and a fluence of 1 × 10^14^ at/cm^2^ at Helmholtz-Zentrum Dresden-Rossendorf, Germany (HZDR). Under these conditions, the calculated Yb ion range was ~30 nm with the maximum of the nuclear energy loss at 20 nm, while the whole thickness of the modified layer reached up to 80 nm [20]. The first part of the samples was implanted at room temperature and then subjected to post-implantation RTA processes. The structural and optical properties of these samples were compared with the properties of the second set of the samples, which were implanted at an elevated temperature (500, 700, and 900 °C). RTA, with an oxygen atmosphere at 800 °C for 1, 5, 10, and 20 min, was performed at the IP PAS using Accu Thermo AW-610 from Allwin21 Co. system (Morgan Hill, CA, USA). Other parameters used for different RTA processes included variations in the temperature (700 °C, 800 °C, and 900 °C) and annealing in argon and nitrogen atmosphere at 800 °C for 10 min. The standard RBS/c measurements were performed with 1.7 MeV He^+^ ions using a Van de Graaff accelerator at Helmholtz-Zentrum Dresden Rossendorf (HZDR), Germany. In the RBS/c experiments, a silicon detector positioned at a scattering angle of 170^o^ was used, with a depth resolution <5 nm and an energy resolution <20 keV. Atomic force microscopy (AFM), Bruker Dimension Icon (Bruker, Santa Clara, CA, USA), using silicon nitride probes with sharp tips (a tip radius: 2 nm) in the Peak Force Tapping mode was employed to image the surface morphology and the roughness (indicated as RMS) at the IP PAS. The RMS value for all samples was detected for scanning areas of 10 × 10 μm. High resolution X-ray diffraction (HRXRD) measurements were performed using a Panalytical X’Pert Pro-MRD diffractometer (Malvern PANanalytical, Westborough, MA, USA) equipped with a Cu anode X-ray lamp, a two-bounce Ge (220) hybrid monochromator, and a three-bounce Ge (220) analyzer placed in front of Pixcel or Proportional detectors. Photoluminescence (PL) spectroscopy measurements of Yb^3+^-implanted β-Ga_2_O_3_ were carried out at T = 300 K using an Edinburgh Instrument FLS 1000 fluorescence spectrometer (Livingston, UK). Yb^3+^-related intra-shell emission spectra of the samples were detected in single photon counting mode in the range 1.25–1.18 eV under near-band edge excitation at 5.17 eV at the maximum of the PL excitation (PLE) spectrum of Yb^3+^ ions.

## 3. Results and Discussion

### 3.1. Crystal Lattice Recovery

**Figure 2:**
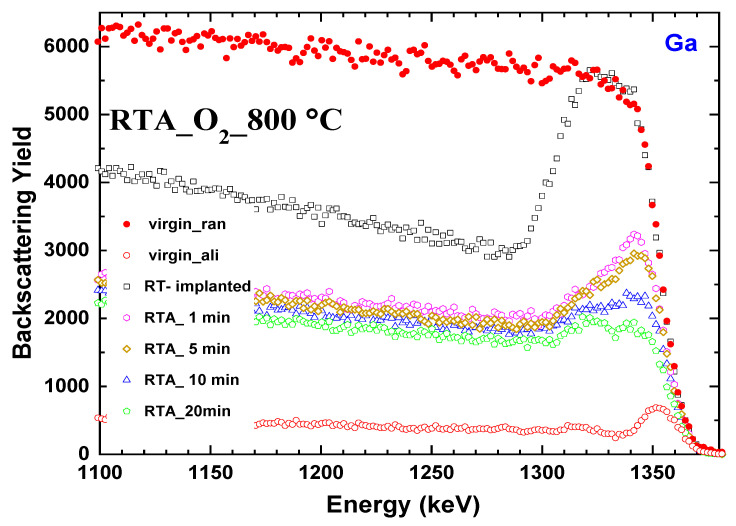
Random (ran, solid symbols) and aligned (ali, open symbols) RBS spectra for β-Ga_2_O_3_ implanted with Yb ions at the fluence of 1 × 10^14^ at/cm^2^ and post-implantation annealed at 800 °C, in O_2_ for different time durations.

Structural changes after annealing performed in various conditions of $\left(\overset{¯}{2}01\right)$-oriented β-Ga_2_O_3_ samples implanted with Yb to the fluence of 1 × 10^14^ Yb ions/cm^2^ were evaluated via RBS/c [25] and supported by McChasy [26,27], the Monte Carlo simulation code. The RBS spectra were collected in two different modes of measurement: random and aligned (Figure 2). Near the crystal surface (1290–1350 eV), the RBS spectrum for the fluence of 1 × 10^14^ Yb ions/cm^2^ is close to the height of the random level that represents the amorphization level (no crystal channels of the matrix are visible). The evolution of the damage peak with an increasing annealing time in an oxygen environment at 800 °C demonstrates that the annealing time is crucial for the crystal lattice recovery. Starting with annealing for 1 min, two defect regions begin to be clearly visible in the damage zone, and the presence of two defect regions has been reported by M. Sarwar et al. [20]. The damage in both regions decreased with the increase in annealing time. However, the damage in the deeper region decreased more rapidly as compared to the one near the surface, which is accompanied by a slight decrease in the dechanneling level too. In addition, the deeper damage peak seems to be less affected by a longer time of annealing than that closer to the surface. This indicates the presence of a different and more annealing-resistant type of defect in the depth. The reduction in the intensity of the surface damage peak with longer annealing time indicates that the oxygen deficiency on the surface of the implanted β-Ga_2_O_3_ diminished with an increased time of exposure to oxygen [28]. In the proposed scenario, the deeper damage region is a mixture of other simple and complex defects; some of them appear to be RTA-resistant [20].

**Figure 3:**
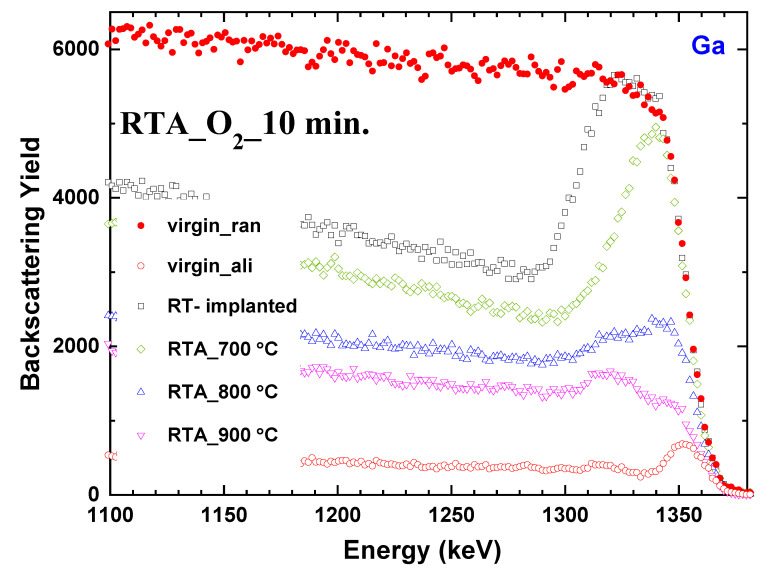
Random (ran, solid symbols) and aligned (ali, open symbols) RBS spectra for β-Ga_2_O_3_ implanted with Yb ions at the fluence of 1 × 10^14^ at/cm^2^ and post-implantation annealed in O_2_ at different temperatures for 10 min.

Similarly to the above experiment, annealing in oxygen at various temperatures (700, 800, and 900 °C) also indicates two damage regions of different origin (Figure 3). As can be seen, the annealing at 700 °C results in a decrease in the thickness of the damage peak as well as the dechanneling level, and there is only a slight change in the intensity of the damage peak present at the surface. It is evident from the RBS spectra that this temperature is not sufficient for the recovery of the implanted β-Ga_2_O_3_ crystals. A further increase in temperature to 800 °C resulted in a significant reduction in the surface damage and revealed two separate damage regions that were not clearly visible for annealing at 700 °C due to the substantial contrast in intensities. The RTA at 900 °C provided further recovery. It is worth noting that the two observed damaged regions reacted differently to temperature changes.

**Figure 4:**
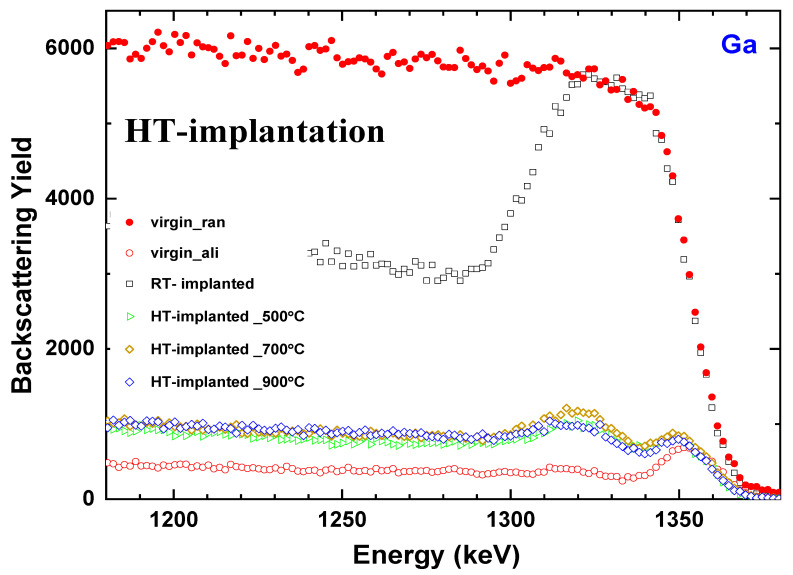
Random (ran, solid symbols) and aligned (ali, open symbols) RBS spectra for β-Ga_2_O_3_ implanted with Yb ions at the fluence of 1 × 10^14^ at/cm^2^ at high temperatures.

The high crystal recovery is validated by the RBS spectra obtained for the HT implantation of β-Ga_2_O_3_:Yb samples performed at various temperatures. However, the implantation temperature, ranging from 500 °C to 900 °C, does not provide significant differences in the recovery level (Figure 4). The aligned spectra presented in Figure 4 show that the surface damage peak for the HT-implanted samples is very close to that of the virgin crystal, and only a slight concentration of defects exists in the deeper region. Comparatively less damage is created in the crystal after HT implantation compared to RT implantation because the majority of the produced point defects are annihilated during HT implantation due to their high mobility.

**Figure 5:**
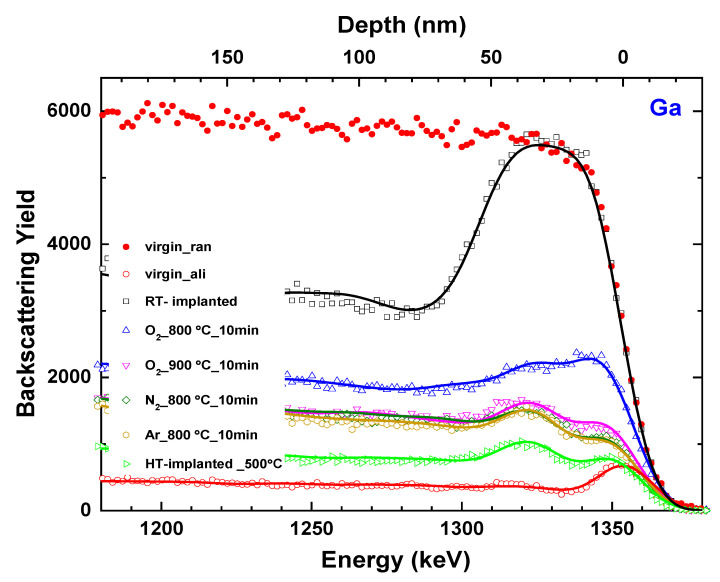
Comparison of various annealing processes. Random (ran, solid symbols) and aligned (ali, open symbols) RBS spectra for β-Ga_2_O_3_ implanted with Yb ions at with the fluence of 1 × 10^14^ at/cm^2^ at HT and RT, and subsequently post-RT implantation annealed at different conditions. The solid line represents the results of the McChasy simulations.

In the last step of this study, the damage behavior of the β-Ga_2_O_3_:Yb crystals annealed in different atmospheres, nitrogen, and argon at 800 °C for 10 min was also tested (Figure 5). The damage profiles reveal that argon and nitrogen annealing leads to a recovery similar to that caused by annealing in oxygen at the highest temperature used (900 °C). However, the lowest level of damage was obtained after the HT implantation.

**Figure 6:**
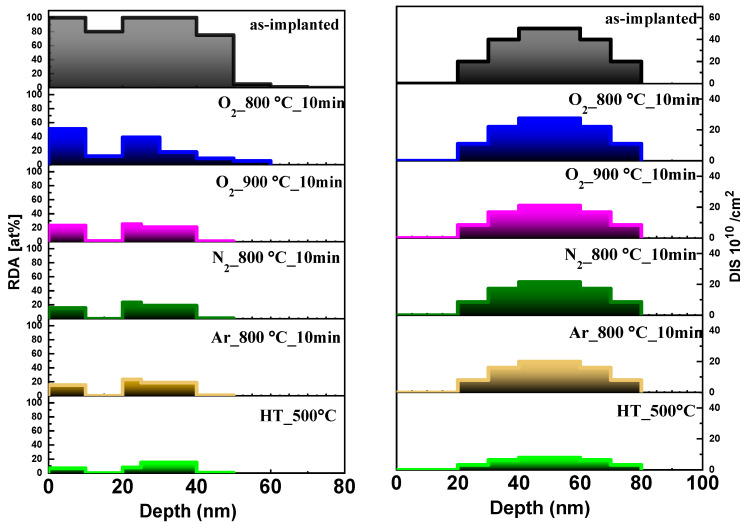
Depth distributions of RDA-type defects and DIS-type defects obtained via McChasy simulations for β-Ga_2_O_3_ implanted with Yb ions with a fluence of 1 × 10^14^ at/cm^2^ at HT and RT, and subsequently post-RT implantation annealed under different conditions.

In order to quantitatively compare various annealing processes, information about the defects structure and their profile was extracted from the McChasy simulations. The solid lines in Figure 5 represent the best fits of the RBS/c spectra obtained with the defect depth distributions presented in Figure 6. As a result of the McChasy simulations, a set of two different defect depth distributions are obtained: depth distribution of RDA-type defects (concentration in at%) and DIS-type defects (concentration in cm^−2^). The RDA-type defect distributions are composed of two damage regions separated by a depleted region that extends from ~10 nm to a depth of ~20 nm, which has become clearly visible for annealed and HT-implanted samples. This region is located at the depth of maximum nuclear energy loss R_pd_ calculated via SRIM, while the deeper defect region overlaps the Yb ions range [20]. As can be deduced from the results presented above, this separation in the defect distribution is due to two different RDA-type defects, as they react differently to annealing. 

Moreover, as can be seen, the RDA-type defects extend to a depth up to ~60 nm, while the DIS-type defect distributions are slightly shifted in depth compared to RDA, beyond the range of the Yb ions. Such a phenomenon of post-implantation damage expanding to a greater depth than that of the implanted ions has been already observed for ZnO [29,30,31] and is explained as the migration of interstitials and vacancies [32]. 

The concentration of both types of defects changes significantly as a result of the thermal treatment. After 10 min of RTA in oxygen at 800 °C, the concentration of RDA-type defects near the surface has decreased from ~100% to ~50%, and the deeper RDA damage region is reduced to ~40%. Annealing in oxygen at 900 °C causes a dramatic change in the RDA distribution near the surface in contrast to the damage in the depth. Annealing in nitrogen and argon provides a similar distribution of both types of defects. The DIS-type defect distribution shows an absence of complex defects near the surface, and their average concentration decreases by a half for all the annealing conditions used. For HT implantation, only a small number of both types of defects are created in the lattice. 

### 3.2. Yb Ion Location

**Figure 7:**
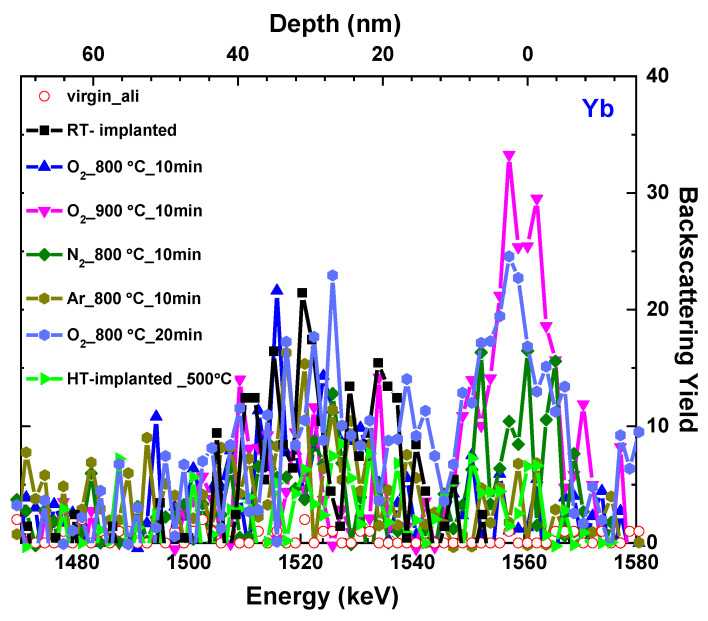
Aligned (ali, solid symbols) RBS spectra for Yb signal of β-Ga_2_O_3_ implanted with Yb ions at the fluence of 1 × 10^14^ at/cm^2^ at HT and RT, and subsequently post-RT implantation annealed at different conditions.

The recovery of the crystal is quite often associated with the out-diffusion of the dopant and its precipitation on the surface, which could cause the dopant to be optically inactive [18,33]. In the case of β-Ga_2_O_3_ implanted with Yb, the out-diffusion process could be easily evaluated based on the RBS spectra as the Yb signal appears separately at the higher energy region (see Figure 7). As can be seen in Figure 6, the highest-used temperature annealing (900 °C) in oxygen led to the precipitation of the Yb on the sample’s surface, visible as a peak around 1560 keV energy region. Also, prolonging the annealing time up to 20 min in oxygen at 800 °C results in Yb precipitation on the surface. In the other tested cases of annealing, the Yb out-diffusion is not evident. 

### 3.3. High-Resolution X-ray Diffraction

**Figure 8:**
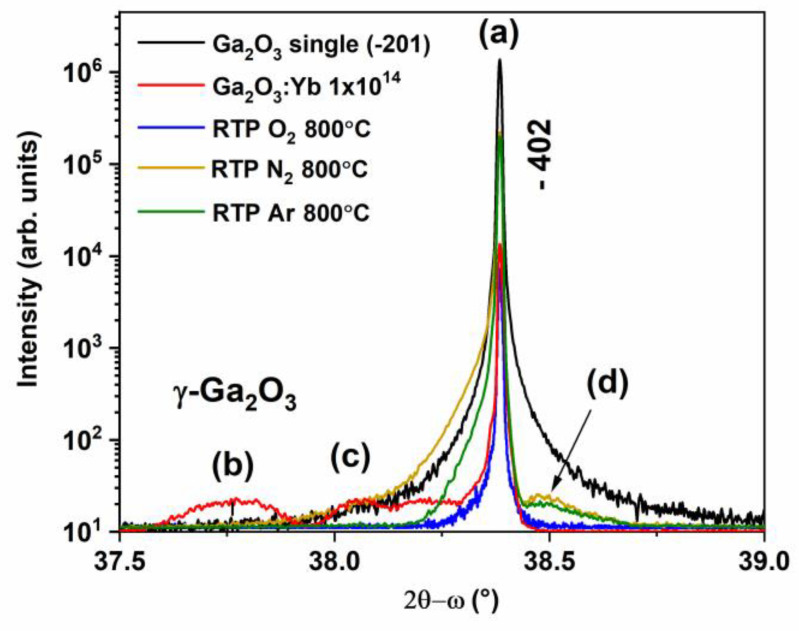
The 2θ-ω scan of the symmetrical −402 reflection of a virgin β-Ga_2_O_3_ crystal (black). This crystal was implanted at RT with Yb ions with a fluence of 1 × 10^14^ at/cm^2^ (red) and subsequently post-RT-implantation annealed for 10 min. at 800 °C in the O_2_ (blue), N_2_ (yellow), and Ar (green) atmosphere.

The 2θ-ω scans were measured for $\left(\overset{¯}{2}01\right)$-oriented β-Ga_2_O_3_ bulk crystals implanted with 1 × 10^14^ at/cm^2^ of Yb and post-implantation annealed for 10 min. in different ambient gases at 800 °C (Figure 8). 

The −402 reflection peak appearing at 38.38° (Figure 8, a peak) with a full width at high maximum (FWHM) of 0.012° confirms the high quality of the single β-Ga_2_O_3_ crystal used in the experiment. After the implantation, an additional broad peak at 37.76° appears and disappears after all three annealing conditions. According to a previous report [20], it can be assigned to the γ-Ga_2_O_3_ phase (Figure 8, (b peak)). Additionally, a shoulder of the main peak that is visible at 38.06^o^ for the as-implanted as well as N_2_- and Ar-annealed crystals (Figure 8, (c peak)) can be assigned to implantation-induced strain [17]. It is worth noting that after oxygen annealing, a sharp peak without any shoulder peak can be observed. However, in the case of nitrogen and argon annealing, a shoulder peak at a higher angle of 38.49° (Figure 8, (d peak)) and (c) peak broadening can be seen and are related to implantation-induced strain.

### 3.4. Surface Morphology

**Figure 9:**
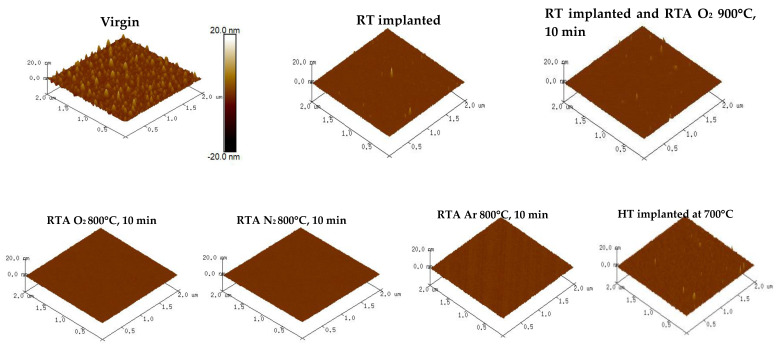
AFM imaging of the surface morphology for virgin β-Ga_2_O_3_, implanted with 150 keV Yb ions to the fluence of 1 × 10^14^ at/cm^2^, RTA-annealed in different conditions, and HT-implanted.

The surface morphology and roughness of the virgin β-Ga_2_O_3_, implanted with Yb and post-implantation annealed in different annealing conditions, was measured via atomic force microscopy (AFM) (Figure 9 and Table 1). The RMS value of the virgin sample is 1.3 nm. The surface became smoother after the implantation with the roughness varying from 0.2 nm to 1.3 nm. The annealing of the implanted β-Ga_2_O_3_ further alleviated the roughness, as can be seen in Table 1, for lower temperature annealing in oxygen at 700 °C for 10 min. A similar effect was reported by M. Sarwar et al. [20]. As shown by unpublished SEM images, the cause is empty tracks present in the pristine crystals and created during polishing, which are filled by annealing. However, the high temperature (800 °C) but shorter time (1 min) did not lead to a smooth surface, but the roughness remained the same as that of the as-implanted sample. It is obvious that the shorter annealing time did not have a significant effect on the surface. Various atmospheres had different effects on the surface with the nitrogen annealing leading to a smoother surface (RMS = ~0.2 nm) than Ar (RMS = ~0.3 nm) and oxygen annealing (RMS = ~0.6 nm) at 800 °C. The higher temperature oxygen annealing (900 °C) results were particularly different. The roughness of the layer is similar to that of the virgin β-Ga_2_O_3_, i.e., 1.3 nm, but it can be seen in the morphological image that the surface looks flatter as compared to the virgin one (top left image in Figure 9). Based on the RBS results, we conclude that it might be due to the creation of nano-sized clusters of Yb ions, which precipitated on the surface.

### 3.5. Room-Temperature Photoluminescence

**Figure 10:**
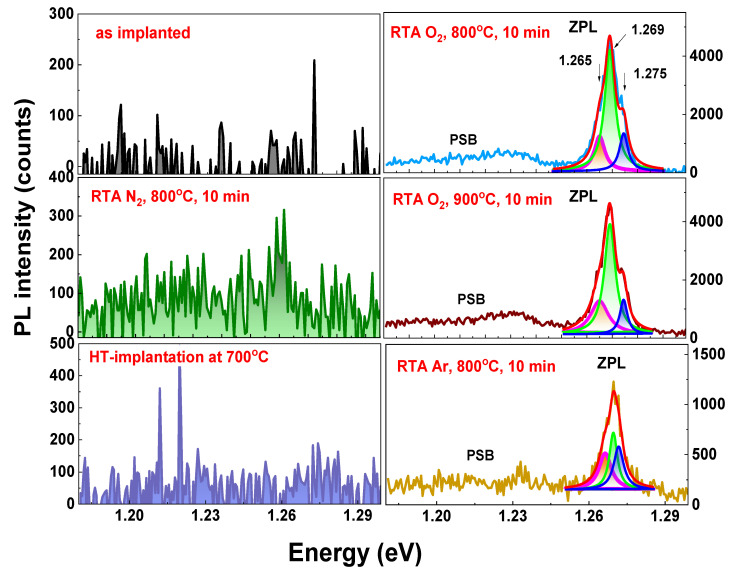
RT PL spectra in IR region obtained for β-Ga_2_O_3_ crystal implanted with Yb ions to a fluence of 1 × 10^14^ at/cm^2^ for as-implanted sample at RT and HT temperatures and for RT-implanted and subsequently RTA-annealed samples for 10 min. at different conditions.

The optical response of Yb-implanted β-Ga_2_O_3_ crystals to different thermal treatment conditions has been studied via photoluminescence (PL) spectroscopy performed at 300 K with an excitation energy of 5.17 eV. As can be seen in Figure 10, there is no luminescence observed in the as-implanted crystal. Also, in the nitrogen-annealed and HT-implanted crystals, which have lower defect concentrations according to RBS/c, the IR luminescence is hardly visible. On the contrary, annealing in oxygen at 800 °C for 10 min led to the intense PL of the characteristic zero phonon line (ZPL) of the intra-shell ^2^*F*_5/2_ → ^2^*F*_7/2_ transition of the Yb^3+^ ion at 1.269 eV (976 nm), which is the main radiative transition of Yb ions in the 3+ charge state [13,34], and its weak phonon side band (PSB) in the range 1.18–1.24 eV, corresponding to the Yb^3+^ ion emission vibronic band (^2^*F*_5/2_→^2^*F*_7/2_(*n*); *n*  =  1, 2, 3, 4) [35], is observed. The ZPL is complex, consisting of three Lorentzian components with energies of 1.265, 1.269, and 1.275 eV. The origin of such a splitting of the ZPL can be explained by the structural defects in the β-Ga_2_O_3_ matrix that can introduce defect states during implantation [36,37,38,39].

High-temperature annealing in oxygen at 900 °C results in a similar Yb^3+^ emission behavior and efficiency. Interestingly, even though the argon annealing at 800 °C directs almost the same recovery as high-temperature annealing in oxygen, the PL intensity of the main emission peak centered at 1.269 eV is about four times lower. 

## 4. Conclusions

The optical response of $\left(\overset{¯}{2}01\right)$-oriented β-Ga_2_O_3_ single crystals implanted with 1 × 10^14^ at/cm^2^ Yb fluence and energy 150 keV was studied after the transformation of crystal lattice defects resulting from post-implantation annealing. Various annealing temperatures, durations, and atmospheres have been tested to monitor both the crystal lattice recovery as well as the location of Yb ions in the matrix in order to find the optimal annealing conditions for a strong optical response, which is crucial for potential optoelectronic applications. 

The presented research has shown that extending the time and higher temperature of annealing in oxygen leads to a reduction in the damage level, which is an indication of lattice recovery. However, when too high a temperature (900 °C) or too long an annealing time (20 min) is used, the out-diffusion of Yb ions to the surface is observed. Moreover, the level of lattice recovery after annealing depends on the annealing atmosphere, and the effect of the annealing carried out in Ar and N_2_ at 800 °C for 10 min is similar to the effect of annealing in O_2_ at 900 °C. 

It is worth noting that in Yb-implanted and post-implantation annealed β-Ga_2_O_3_, two separate regions of RDA-type defects appear, which respond differently under different annealing conditions. This indicates that two types of RDA-type defects are formed during the implantation of β-Ga_2_O_3_ single crystals. Furthermore, the high-temperature implantation performed at temperatures ranging from 500 °C to 900 °C was shown to be an alternative way to eliminate defects and Yb ion out-diffusion, which provides the prospect of gallium oxide implantation with less crystal damage and the efficient incorporation of the RE dopant into the matrix. 

The IR luminescence results have shown the strongest optical response of Yb for the oxygen-annealed crystals, while for the argon-annealed samples, the optical response was four times lower. For both the RT- and HT-implanted samples, as well as RT-implanted and nitrogen-annealed samples, the Yb^3+^ emission is almost absent. This shows that crystal lattice recovery, which is perfect for HT implantation, is not a sufficient condition for achieving a strong optical response from the β-Ga_2_O_3_:Yb system and suggests an important role of oxygen in activating the RE ion luminescence.

## Figures and Tables

**Table 1:** RMS value from the AFM imaging of the surface morphology for virgin β-Ga_2_O_3_, implanted with 150 keV Yb ions to the fluence of 1 × 10^14^ at/cm^2^, RTA-annealed in different conditions and HT-implanted.

Different RTA Temperatures		Different RTA Time		Different RTA Atmospheres		HT Implantation	
Sample	RMS (nm)	Sample	RMS (nm)	Sample	RMS (nm)	Sample	RMS (nm)
Virgin	1.3	O_2_ 800 °C, 1 min	0.7	N_2_ 800 °C, 10 min	0.2	500 °C	0.2
RT implanted	0.7	O_2_ 800 °C, 5 min	0.6	Ar 800 °C, 10 min	0.3	700 °C	1.0
O_2_ 700 °C, 10 min	0.2	O_2_ 800 °C, 10 min	0.6	O_2_ 800 °C, 10 min	0.6	900 °C	0.3
O_2_ 800 °C, 10 min	0.6	O_2_ 800 °C, 20 min	0.3	-	-	-	-
O_2_ 900 °C, 10 min	1.3	-	-	-	-	-	-

## Data Availability

The original contributions presented in the study are included in the article, further inquiries can be directed to the corresponding author.
